# Classification of Pediatric Gangliogliomas Based on the Histological Infiltration

**DOI:** 10.3390/curroncol29100532

**Published:** 2022-09-21

**Authors:** Murad Alturkustani

**Affiliations:** Department of Pathology, Faculty of Medicine, King Abdulaziz University, Jeddah 21589, Saudi Arabia; alturkustani.murad@gmail.com

**Keywords:** ganglioglioma, glioneuronal tumor, infiltrative glioneuronal tumor, diffuse glioneuronal tumor, CNS, tumor

## Abstract

Ganglioglioma is a well-circumscribed low-grade glioneuronal tumor with a broad morphological spectrum. Diffuse glioneuronal tumors are used to describe cases with infiltrative growth. Molecular studies of some of these cases are consistent with ganglioglioma. This work aimed to clarify the growth patterns in ganglioglioma. The available slides and clinical and molecular information for 46 patients (50 samples) with a diagnosis of ganglioglioma under the open pediatric brain tumor atlas from the children’s brain tumor network database were reviewed to confirm the integrated diagnosis and to evaluate the growth patterns in these cases. Ten samples from nine patients were excluded as no slides were available, the integrated diagnoses were changed in seven cases (nine samples), ten cases (ten samples) were diagnosed as low-grade glial/glioneuronal tumors, and the diagnosis of ganglioglioma was confirmed in seventeen samples from sixteen patients (nine females and seven males; age ranges from eight months–19 years with a mean of 9.9 years). Infiltration is defined as the presence of neoplastic cells among the nonneoplastic parenchyma. The growth pattern was predominantly circumscribed in six cases, predominantly infiltrative in five cases, and combined growth patterns in five cases. This work confirmed the presence of an infiltrative/diffuse variant of ganglioglioma as a significant pattern. The differential diagnosis in these cases was mainly infiltrative glioma, usually *IDH*-wild type in this population, which may introduce a high-grade glioma in the differential. Awareness of infiltrative ganglioglioma variants should be helpful in this scenario.

## 1. Introduction

Ganglioglioma is a central nervous system (CNS) World Health Organization (WHO) grade 1 biphasic tumor that combines neoplastic glial and neuronal components [1]. It can affect patients of a wide range of ages but most commonly occurs in patients younger than 20 years, and it is the most common tumor associated with long-term epilepsy [2,3].

The essential morphological features to diagnose a ganglioglioma are low-grade intra-axial tumors with a combination of neoplastic neurons and glial cells. The definition of neoplastic neurons includes both dysmorphic/dysplastic neurons and neurons with near-normal morphology [1]. The glial component may resemble pilocytic astrocytoma, oligodendroglioma, and fibrillary astrocytoma [4]. This definition creates a broad spectrum of histological features that fulfill the diagnostic criteria of ganglioglioma.

Ganglioglioma is a relatively circumscribed tumor, but the literature has documented some cases with infiltrative growth patterns [1]. However, diffuse growth is not well defined, and when encountered, it creates doubt about the accuracy of the diagnosis. These cases may be diagnosed as diffuse glioneuronal tumors [5], and molecular alterations in some cases support ganglioglioma diagnosis [5]. The differential diagnosis of ganglioglioma with infiltration will include other forms of glioneuronal tumors and infiltrative gliomas depending on the appreciation of the neuronal component as a part of the tumor in the former or as a preexisting component in the latter.

The distinction of ganglioglioma from other glioneuronal tumors may have minor consequences, as most represent central nervous system (CNS) World Health Organization (WHO) grade 1 tumors. These tumors include dysembryoplastic neuroepithelial tumor (DNT) [5], polymorphous low-grade neuroepithelial tumor of the young (PLNTY) [6], and multinodular and vacuolating neuronal tumor (MNVT) [7]. However, the distinction from infiltrative glioma is essential, as these tumors will represent isocitrate dehydrogenase (*IDH*)-wild-type tumors, and the differential may include high-grade glioma.

This work aims to expand the morphological spectrum of gangliogliomas in children and clarify the histological diffuse/infiltrative variant of ganglioglioma.

## 2. Materials and Methods

The project proposal was approved by the Children’s Brain Tumor Network (CBTN) (https://cbttc.org, accessed on 1 July 2022). Consent from all patients was obtained per the CBTN protocol. The clinical and processed molecular data are available from PedcBioPortal (https://pedcbioportal.org, accessed on 1 July 2022). The dataset for this project was under the Open Pediatric Brain Tumor Atlas (OpenPBTA). The study group was all cases with the diagnosis of ganglioglioma under the histology section. The scanned pathology slides and the pathology report of selected cases in this group were available for me through the CAVATICA website (https://www.cavatica.org, accessed on 1 July 2022).

There were 50 samples from 46 patients under the histological diagnosis of ganglioglioma. The inclusion criteria were the histological diagnosis of ganglioglioma and the availability of scanned histological slides for evaluation. In addition, the following information was obtained from the clinical and genetic data: age, sex, location of the tumor, and significant genetic alterations.

The pathological features were obtained from the available pathological reports and the examination of the scanned images of the cases. The cases were divided into three groups depending on the final integrated diagnosis: (1) Specific entity other than ganglioglioma. (2) Ganglioglioma when the morphological and molecular alterations of the glioneuronal tumor do not suggest another entity. (3) Glial/glioneuronal tumor if another differential diagnosis cannot be excluded based on the morphological and molecular alterations.

Cases diagnosed with ganglioglioma were then categorized according to the infiltrative-look histologically into relatively circumscribed, infiltrative-looking, and combined. In addition, the following features were assessed in all cases: dysmorphic ganglion cells, eosinophilic granular bodies and Rosenthal fibers, the morphology of the glial component, and molecular alterations.

The infiltrative pattern was defined as the presence of neoplastic cells among the nonneoplastic brain parenchyma. Focal limited infiltration was accepted under the relatively well-circumscribed category. Dysmorphic ganglion cells were defined as ganglion cells with cytoplasmic enlargement, abnormal Nissl cytoplasmic distribution, abnormal neuronal clusters or orientation, and/or the presence of binucleated forms. The degree of dysmorphic ganglion cells was then categorized into those with near-normal neuronal morphology (score 1), scattered dysmorphic ganglion cells (score 2), and frequent dysmorphic ganglion cells (score 3). Finally, the glial component was categorized into pilocytic astrocytoma-like, oligodendroglioma-like, infiltrative astrocytoma-like, or nonspecific glial.

## 3. Results

**Figure 1:**
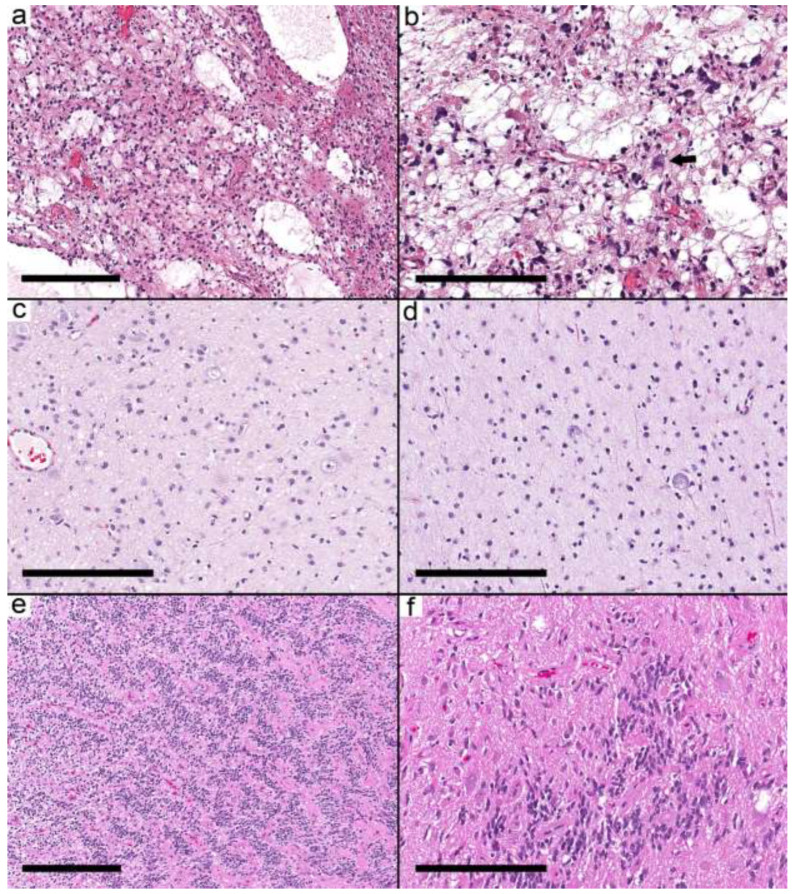
Integrated diagnosis is a specific entity other than ganglioglioma. (**a**) Pilocytic astrocytoma (Case 2) with a biphasic pattern. (**b**) Microcystic space with pleomorphic cells and eosinophilic granular bodies. A single dysmorphic ganglion (arrow) is present. (**c**,**d**) MYB-altered glioma (Case 3) with small round cells and scattered large ganglion cells. (**e**) CNS embryonal tumor (EWSR1-PLAGL1) NEC (Case 5) showing ribbons and trabecular arrangement of small primitive cells and perivascular pseudorosettes. (**f**) Areas with dysmorphic ganglion cells. Scale bars: 600 µm (**a**,**e**); 200 µm (**b**–**d**,**f**).

Fifty samples were available for the study, and forty samples from 37 patients met the inclusion criteria (Supplementary Table S1). The histological and molecular alterations were sufficient in seven cases (nine samples) to change the integrated diagnosis into another well-defined entity (Supplementary Table S1). These were *IDH* mutant astrocytoma, pilocytic astrocytoma (Figure 1a,b), diffuse astrocytoma, MYB-altered, infant-type hemispheric glioma (Figure 1c,d), CNS embryonal tumor, not elsewhere classified (NEC) (Figure 1e,f), MVNT, and PXA. The integrated diagnoses in the remaining cases were ganglioglioma (Supplementary Table S2) in 16 patients (17 samples), desmoplastic infantile ganglioglioma (DIG) in four cases (4 samples), and a low-grade glial/glioneuronal tumor in 10 patients (10 samples) Supplementary Table S3.

The 16 ganglioglioma cases were in nine females and seven males. The ages of patients ranged from 8 months to 19 years, with a mean of 9.9 years. The morphological features can be separated into three groups based on the circumscription of the tumor: infiltrative-looking tumors, relatively circumscribed tumors, and combined infiltrative and circumscribed areas.

**Figure 2:**
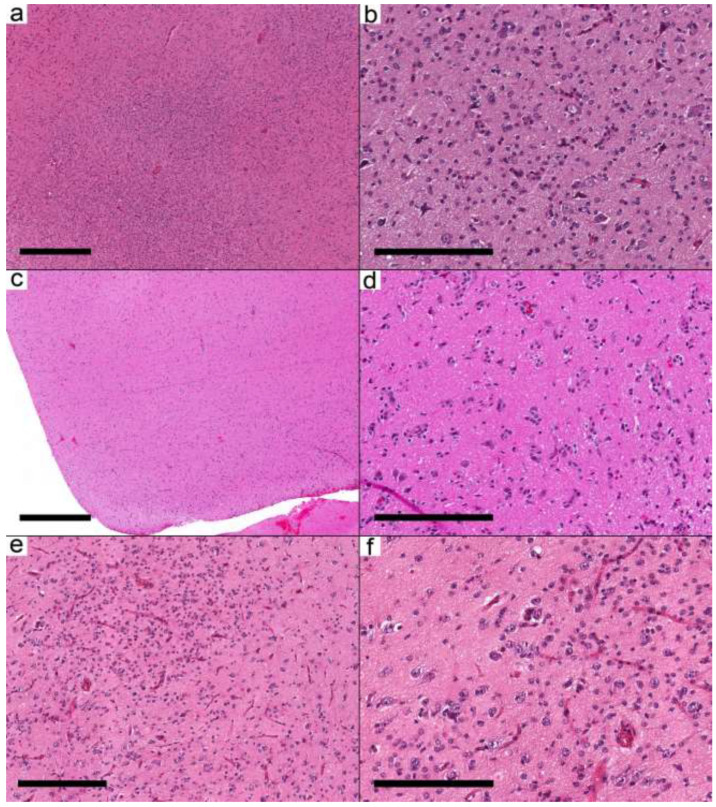
Ganglioglioma with predominant infiltrative pattern. (**a**–**e**) Three different cases show similar morphological features. (**a**,**c**,**e**) The cortical areas are involved by a low-moderately cellular neoplasm formed by dysmorphic neurons and oligodendrocyte-like cells. (**b**,**d**,**f**) Oligodendrocyte-like cells are arranged around neurons and blood vessels resembling infiltrative glioma. Cases: (**a**,**b**) Case 8; (**c**,**d**) Case 9; (**e**,**f**) Case 10. Scale bars: 600 µm (**a**,**c**); 200 µm (**b**,**d**,**f**); 300 µm (**e**).

In the first group (six samples from five cases), the infiltrative nature was mainly in the form of cortical involvement (Figure 2a–e). The cortical areas were involved with low to moderate cellular neoplasms formed by glial cells and dysmorphic ganglion cells. The infiltrative glial cells were arranged around the neurons and the blood vessels, resembling the pattern of infiltrative glioma. The glial background in this group was oligodendroglioma-like in three cases and nonspecific in three samples from two cases. Eosinophilic granular bodies (EGBs) were present in 3/6 samples (2/5 cases). The presence of frequent dysmorphic ganglion cells (score 3), except for Case 8, in these samples supported the diagnosis of ganglioglioma. Case 8 had near-normal appearing neurons. However, due to the location of the tumor in the temporal lobe and the presence of *BRAF* V600E, the integrated diagnosis was ganglioglioma. Case 12 had two samples. The first was taken at the age of 10.7 years and had *KRAS* Q61K and *IDH* R132H with a low allelic frequency (AF) of 0.16. The second specimen was taken at the age of 17 years and showed the *KRAS* Q61K mutation, while *IDH* mutation was not present. Given the frequent dysmorphic ganglion cells, the low *IDH* R132H AF, and the presence of *KRAS* mutation, the integrated diagnosis was considered ganglioglioma rather than *IDH*-mutant glioma.

**Figure 3:**
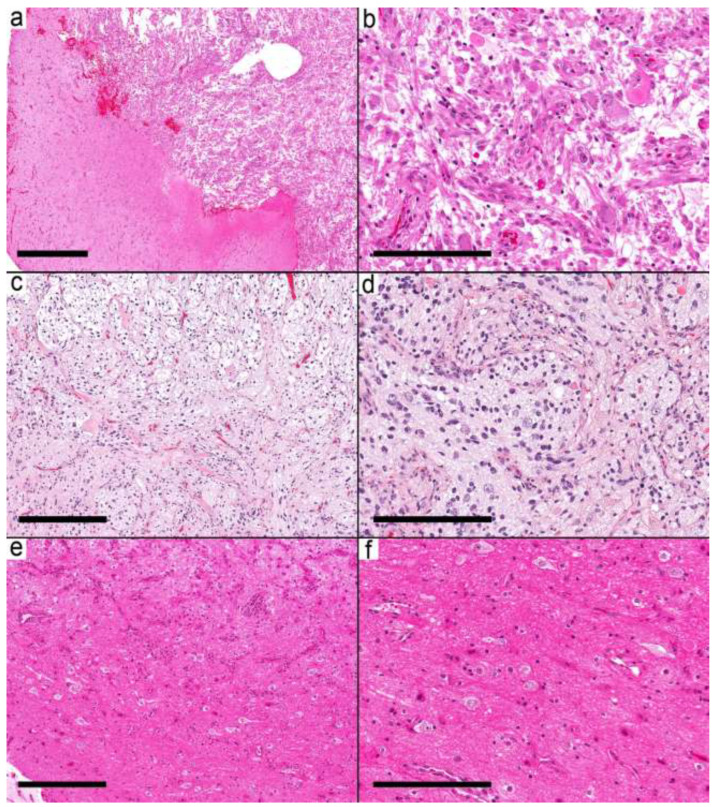
Ganglioglioma with circumscribed pattern. (**a**) Relatively demarcated nodular neoplasm (Case 14) on the right and gliotic brain tissue on the left side. (**b**) The nodule is formed by frequent dysmorphic ganglion cells and few glial cells. (**c**) Relatively demarcated nodule (Case 15) formed by biphasic tumor with microcystic spaces and fibrillary areas resembling pilocytic astrocytoma. (**d**) Frequent dysmorphic ganglion cells and background of the neuropil. (**e**,**f**) Relatively demarcated area (Case 17) with a moderate number of dysmorphic ganglion cells and a minor glial component in the background. Scale bars: 600 µm (**a**); 200 µm (**b**,**d**,**f**); 300 µm (**c**,**e**).

The second group (six samples from six cases) was characterized by relatively well-defined nodules (Figure 3a–f). The nodule was formed by frequent dysmorphic ganglion cells and few glial cells. Most of the glial background in this group was pilocytic astrocytoma-like (three cases), while two cases had nonspecific glia and one had an oligodendroglioma-like pattern. EGBs were present in 5/6 cases; the last case had Rosenthal fibers.

**Figure 4:**
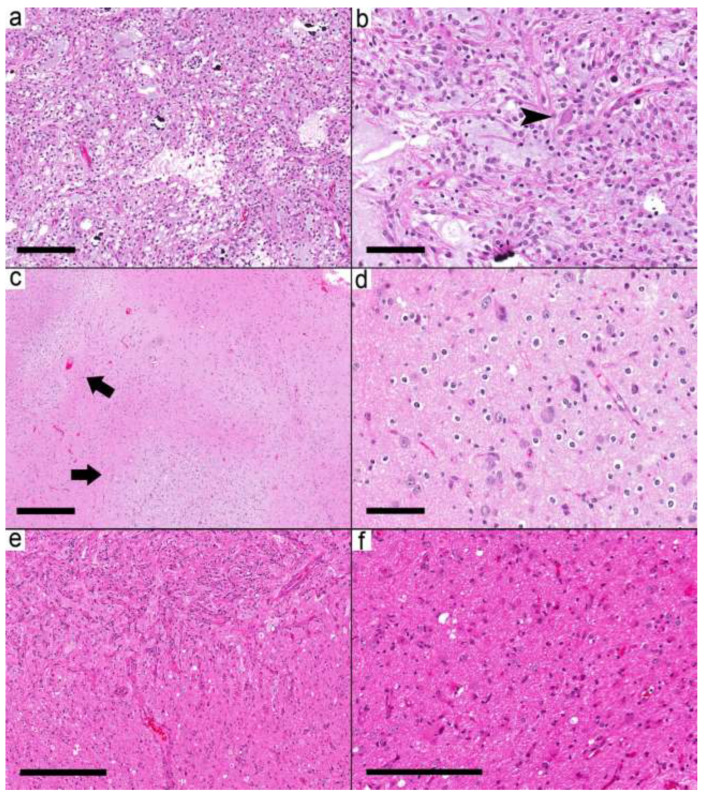
Ganglioglioma with combined circumscribed and infiltrative tumors. (**a**) The relatively demarcated area (Case 19) is formed by microcystic areas and round glial cells. (**b**) Scattered dysmorphic ganglion (arrowhead) in the tumor. (**c**–**f**) Case 20 shows multiple small nodules (arrows). (**d**) These small nodules are formed by frequent dysmorphic ganglion cells and oligodendrocytes in the background. (**e**) A relatively demarcated cellular area with many dysmorphic ganglion cells in the upper left is separated by less-cellular areas with many dysmorphic ganglion cells from the area in (**f**). (**f**) The adjacent area shows a mixture of reactive astrocyte-looking cells and a few dysmorphic ganglion cells. Scale bars: 200 µm (**a**,**f**); 100 µm (**b**,**d**); 500 µm (**c**); 300 µm (**e**).

The third group (five samples from five cases) showed both relatively circumscribed nodules and infiltrative-looking areas (Figure 4a–f). The glial background in this group was pilocytic astrocytoma-like in two cases, nonspecific glial in two cases, and oligodendroglioma-like in one case. EGBs were present in 2/5 cases. Case 19 had a pilocytic astrocytoma-like glial component and KIAA1549-BRAF fusion. However, the presence of frequent dysmorphic ganglion cells, the location of the tumor in the temporal lobe, and the lack of EGBs favored the diagnosis of ganglioglioma over pilocytic astrocytoma.

*BRAF* V600E was the most common alteration in ganglioglioma (6/17 cases) and was distributed among the different morphological groups: two cases in the first group, three cases in the second group, and a single case in the third group.

Four cases showed the morphological features of ganglioglioma but also had areas of desmoplasia, and the affected patients were younger than four years. The differential diagnosis in these cases included desmoplastic infantile ganglioglioma. Next-generation sequencing results were available in three cases, and all had *BRAF* V600E. The integrated diagnosis of DIG was favored in these cases.

**Figure 5:**
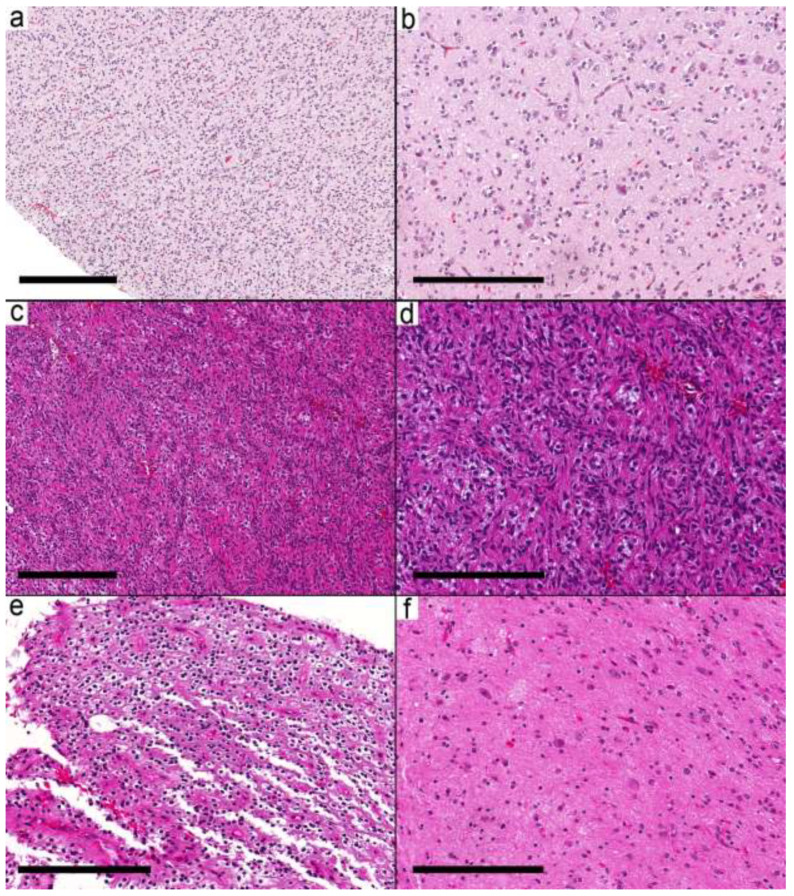
Low-grade glial/glioneuronal tumor. (**a**,**b**) Case 29 shows a cortex with subtle near-normal appearing neurons and infiltrative-looking oligodendrocyte-like cells similar to the infiltrative pattern in ganglioglioma. (**c**,**d**) Case 32 shows a cortex infiltrated by elongated cells with a prominent perivascular arrangement that resembles angiocentric glioma. (**e**) Case 33 showing areas with oligodendrocyte-like cells with perinuclear halos and prominent cell membranes. (**f**) Adjacent focus shows scattered dysmorphic ganglion cells and a nonspecific glial background. Scale bars: 300 µm (**a**,**c**); 200 µm (**b**,**d**–**f**).

In 10 cases, the morphological features and molecular alterations brought another differential diagnosis that could not be excluded from the available information. In these cases, the diagnosis of a low-grade glial/glioneuronal tumor is favored to reflect the uncertainty and difficulty of distinguishing ganglioglioma from its mimickers. The differential was infiltrative glioma in cases 28–31 (Figure 5a,b). In case 32, the histology was suggestive of angiocentric glioma (Figure 5c,d) but showed *KRAS* G12D. The histological features for the following cases suggest the diagnosis in brackets: Cases 33 and 34 (PLNTY, Figure 5e,f), Case 35 (MVNT), Case 36 (rosette-forming glioneuronal tumor (RFGNT)), and Case 37 (DNT). In a clinical setting with the availability of other details and immunostains, most of these cases could have been assigned a specific diagnosis.

## 4. Discussion

Gangliogliomas are better known as circumscribed tumors. However, the essential diagnostic criteria for ganglioglioma in the WHO fifth edition included a wide range of morphological patterns [1]. These histological features are shared by many low-grade neuroepithelial tumors and low-grade infiltrative astrocytoma. As these cases also share similar clinical presentations, molecular alterations, and prognoses, it may be challenging to distinguish between them.

To simplify the morphological spectrum of gangliogliomas, this work categorized the tumor based on their infiltrative pattern histologically into those with an infiltrative look, relatively well-circumscribed, and combined growth patterns. Most gangliogliomas show molecular evidence of activation of the mitogen-activated protein kinase (MAPK) pathway, with the *BRAF* V600E as the most common alteration [8], and the most common chromosomal alteration is the gain of chromosome 7 [9]. However, cases with classic morphological features do not require molecular confirmation [1].

The glial component in ganglioglioma is quite variable. Earlier studies of 61 gangliogliomas documented astrocytoma in 52/61 (85%) as the most common glial component in ganglioglioma, and in the remaining nine cases, areas of pure neoplastic astrocytes were present. The astrocytic component resembled pilocytic astrocytoma in 14 cases, but only one case showed prominent Rosenthal fibers. The neuronal density was sparse in 21 cases, intermediate in 23 cases, and dense in 17 cases [10]. In a larger review of 326 gangliogliomas, oligodendroglioma was added as a possible glial component [11], and rarely, PXA represented the glial component [12].

This study confirmed that histological evidence of infiltration is common in ganglioglioma either as the prominent pattern in 5/16 gangliogliomas or combined with a circumscribed nodule in another five cases. The infiltrative portion mainly involved the cortex. The neoplastic glial cells were in between normal-appearing neurons and mixed with dysmorphic ganglion cells. Similar cases were described in the literature and were called diffuse glioneuronal tumors. The molecular alterations of these tumors were consistent with ganglioglioma [5]. Another form of infiltration better described in the literature is the presence of clusters of neoplastic cells in the cortex adjacent to the primary tumor, which is better appreciated with CD34 immunostain. These were frequent findings in a systematic review of 326 gangliogliomas [11].

The differential diagnosis between ganglioglioma with diffuse infiltration and infiltrative glioma relies on the intermingled neurons as either neoplastic in ganglioglioma or as preexisting neurons in astrocytoma and oligodendroglioma [11]. The challenge in the diagnosis is evident as in a previous study, 14 diffuse astrocytomas and 11 oligodendrogliomas were reclassified as ganglioglioma [4]. Neuronal markers could highlight dysmorphic neurons, as they tend to show no or weak staining for Neu N and perimembranous staining for synaptophysin [1]. CD34 is also immunopositive in glioneuronal tumors and immunonegative in infiltrative astrocytoma. *IDH* mutation is specific for *IDH*-mutant astrocytoma; however, pediatric-type astrocytomas are usually *IDH*-wild type [13].

The *BRAF* V600E mutation detected by immunostaining or molecular studies is expected in ganglioglioma but can be positive in many other tumors, including low-grade glioma, PXA, DNT, and other glioneuronal tumors [14]. In ganglioglioma, *BRAF* V600E immunoreactivity can be restricted to dysmorphic ganglion cells (16/41 cases); however, glial cells and intermediate cells with neuronal and glial differentiation can be immunopositive [15]. In addition, this mutation is thought to be associated with epileptogenicity [16] of the tumor.

The other differential diagnoses included cases that were either reclassified or suggested as an alternative diagnosis in this cohort. These cases were pilocytic astrocytoma, MVNT, PXA, PLNTY, RFGNT, angiocentric glioma, and DNT. The latter represents a challenging differential, as many previous studies showed significant overlap between these two entities with a poor interobserver agreement in the histological diagnosis among experts [5]. However, molecular studies using either RNA expression or methylation profiles showed promising results in separating these entities into two different groups [17]. The first group is associated with BRAF alterations, CD34 immunostaining, and astrocytic cells as the glial component, while the second group is associated with FGFR1 alteration, immunonegative CD34, and oligodendroglia-like cells as the glial component [17].

The second morphological pattern represents the typical ganglioglioma with a relatively well-circumscribed nodule containing frequent dysmorphic neurons. The differential diagnosis for this pattern includes well-circumscribed tumors such as pilocytic astrocytoma and DNT. Pilocytic astrocytoma is a challenging differential, as it considerably overlaps with ganglioglioma histologically, radiologically [18] and molecular alterations in both tumors resulted in the activation of the MAPK pathway. This challenge is evident in earlier studies, as 25 cases diagnosed as pilocytic astrocytomas were reclassified as ganglioglioma [4]. In the temporal lobe, many pilocytic astrocytomas recurred with features diagnostic of ganglioglioma [19]. However, in pediatric cerebellar tumors with morphological features of pilocytic astrocytoma and foci of dysmorphic ganglion cells and *KIAA1549-BRAF* fusion, Gupta et al. recommended the diagnosis of pilocytic astrocytoma with gangliocytic differentiation instead of ganglioglioma [20]. Following this recommendation, the diagnosis of Case 2 was changed from ganglioglioma to pilocytic astrocytoma.

Other differential diagnoses include high-grade tumors. The possibility of anaplastic ganglioglioma may require more studies, as most previous studies did not include molecular alterations in the described tumors [1], and when available, it either shows no significant alteration [21] or alterations diagnostic of another entity [22]. The anaplastic ganglioglioma in this cohort (Case 5) showed EWSR1-PLAGL1. A recent study suggested that these tumors may represent a unique, different entity [23] and may be better not considered anaplastic ganglioglioma. Another setting would be if diffuse astrocytoma was the alternative diagnosis and *IDH* was wild type. Molecular alterations may be necessary in these cases to exclude the possibility of glioblastoma.

The limitation of this study is its retrospective nature, which depends on the available information in the database. Certain helpful immunostains were not available. The 10 cases with the diagnosis of low-grade glial/glioneuronal tumors could have been assigned specific diagnoses if this information were available. This study also highlights an important aspect when dealing with such a database. The histological diagnoses in these databases usually do not reflect the recent advances in classifying CNS tumors, including the newly recognized entities. Many diagnoses, as demonstrated here, could have changed to better integrated diagnoses. In the current study, 7 cases were rediagnosed as new entities, and ten more could potentially have new diagnoses. Studies that depend on the pathology in these databases should confirm the pathological diagnosis as CNS WHO classification keeps evolving with new entities and integrated molecular findings.

## 5. Conclusions

In conclusion, this study confirmed/clarified the infiltrative/diffuse variant in ganglioglioma among the morphological spectrum of ganglioglioma. The high percentage of this pattern among the cases suggests that this pattern should be considered a classic for ganglioglioma. However, the possibility of diffuse glioma should be excluded before making this diagnosis. In case of doubt, molecular studies could be performed, and if not conclusive, a general terminology such as low-grade glial/glioneuronal tumor could be used.

## Data Availability

All relevant data files are available from the corresponding author (MA) upon request.

## Supplementary Materials

The following supporting information can be downloaded at: https://www.mdpi.com/article/10.3390/curroncol29100532/s1, Table S1: Clinical and pathological features of cases with different integrated diagnosis; Table S2: Clinical and pathological features of cases with integrated diagnosis of ganglioglioma; Table S3: Clinical and pathological features of cases with integrated diagnosis of other glial/glioneuronal tumors.

## Abbreviations

CBTN: children’s brain tumor network; CD: cluster of differentiation; CNS: central nervous system; DIG: desmoplastic infantile ganglioglioma; DNT: dysembryoplastic neuroepithelial tumor; EGD: eosinophilic granular bodies; IDH: isocitrate dehydrogenase; MAPK: mitogen-activated protein kinase; MVNT: multinodular and vacuolating neuronal tumor; NEC: not elsewhere classified; NOS: not otherwise classified; PBTA: pediatric brain tumor atlas; PLNTY: Polymorphous low-grade neuroepithelial tumor of the young; PXA: pleomorphic xanthoastrocytoma; RFGNT: rosette-forming glioneuronal tumor; WHO: World Health Organization.
